# Toward Meeting the Needs of Homeless People with Schizophrenia: The Validity of Quality of Life Measurement

**DOI:** 10.1371/journal.pone.0079677

**Published:** 2013-10-25

**Authors:** Pascal Auquier, Aurelie Tinland, Cecile Fortanier, Anderson Loundou, Karine Baumstarck, Christophe Lancon, Laurent Boyer

**Affiliations:** 1 Aix-Marseille University, EA 3279 Research Unit, Marseille, France; 2 Department of Psychiatry, Sainte-Marguerite University Hospital, Marseille, France; 3 Department of Public Health, University Hospital, Marseille, France; Baylor College of Medicine, United States of America

## Abstract

**Objective:**

To provide new evidence regarding the suitability of using quality of life (QoL) measurements in homeless people with schizophrenia, we assess the acceptability and psychometric properties of a specific QoL instrument (S-QoL 18) in a population of homeless people with schizophrenia, and we compare their QoL levels with those observed in non-homeless people with schizophrenia.

**Methods:**

This multi-centre prospective study was conducted in the following 4 French cities: Lille, Marseille, Paris and Toulouse. Two hundred and thirty-six homeless patients with schizophrenia were recruited over a 12 month-period. The S-QoL 18 was tested for construct validity, reliability, external validity and sensitivity to change. The QoL of the 236 homeless patients was compared with 236 French age- and sex-matched non-homeless patients with schizophrenia.

**Results:**

The eight-factor structure of the S-QoL 18 was confirmed by confirmatory factor analysis (RMSEA = 0.035, CFI = 0.95, GFI = 0.99 and SRMR = 0.015). Internal consistency, reliability and sensitivity to change were satisfactory. External validity was confirmed via correlations between S-QoL 18 dimension scores and SF-36, symptomatology and recovery scores. The percentage of missing data did not exceed 5%. Finally, homeless patients had significantly lower QoL levels than non-homeless patients with schizophrenia.

**Conclusions:**

These results demonstrate the satisfactory acceptability and psychometric properties of the S-QoL 18, suggesting the validity of QoL measurement among homeless patients with schizophrenia. Our study also reported that QoL levels in homeless patients with schizophrenia were dramatically low, highlighting the need for new policies to eradicate homelessness and tackle poverty.

## Introduction

Homelessness is an increasing problem among people living with schizophrenia [1–3]. Schizophrenia is over-represented in homeless populations when compared to non-homeless populations. Indeed, the prevalence of the condition is estimated to be 11% (range 4–16%) [4]. The management of patients with schizophrenia is particularly challenging because this sub-population of the homeless is among the most vulnerable and hardest to reach [5,6]. This group has multiple health problems, including alcohol and substance abuse disorders as well as chronic illnesses, e.g., human immunodeficiency virus, hypertension, and diabetes [7]. Treatment adherence and continuity of care in this population tends to be quite poor, and the already limited access to appropriate care [8] noted in this population is exacerbated by self-neglect and fear of being institutionalised [9]. They also have greater problems with employment, social relationships and family relationships than homeless people who do not suffer from mental illness [10–12]. 

Accurate and appropriate assessment of health status is critical to determining the efficacy of treatment and more globally to medico-social programs and policies among homeless patients with schizophrenia. 

One difficulty is the presence of relevant indicators that take into account the complexity of these populations’ health problems and needs. Quality of life (QoL) measurements are of the utmost importance for evaluating treatment and managing care in patients with schizophrenia and offer a more global and comprehensive assessment of health status than traditional indicators (i.e., symptomatology scales) [13–16]. QoL might encompass numerous dimensions for homeless patients with schizophrenia, e.g., psychological status, functional abilities, personal well-being, social interaction, economic status, vocational status and physical health [17]. 

However, QoL measures have been rarely validated with homeless populations [18]. The limited access to care of homeless explains that they are not well represented in validation studies conducted in health care settings. Moreover, the lack of insurance coverage of homeless does not allow them to participate to studies in some countries. The extent to which QoL measurement remains relevant and valid for homeless patients is a crucial issue that has been insufficiently examined. 

To our knowledge, only two studies have explored this issue in homeless people [19,20]. These studies provided evidence that QoL questionnaires could be reliable/valid measures of health status among the homeless. However, these studies 1) did not specifically consider homeless patients with schizophrenia (only 18 individuals of the 250 studied suffered from schizophrenia in the study by Garcia-Rea et al. [19]); 2) studied generic QoL questionnaires (WHOQOL-100 [19] and SF-36 [20]); 3) did not report how the factorial structure described in the sample fit with the initial structure of the tested instrument, which is a key point when considering validity in these specific populations, who were not included in the development of questionnaires [21,22]; and 4) did not explore the sensitivity to change of the instruments, which is one of the most essential requirements of an outcome measure [13,23].

In this study, we hope to provide new data regarding the suitability of QoL measurements among homeless people with schizophrenia. To this end, we propose to assess the acceptability and the psychometric properties of a specific QoL instrument (the Schizophrenia - Quality of Life short-version questionnaire: S-QoL 18 [13,24]) in a population of homeless patients with schizophrenia and we compare their QoL levels with those observed in non-homeless people with schizophrenia.

## Methods

### Study design and population

This multi-centre prospective study was conducted in the following 4 French large cities: Lille, Marseille, Paris and Toulouse. The inclusion criteria were as follows: age over 18 years; absolutely homeless (i.e., no fixed place to stay for at least the past 7 nights with little likelihood of finding a place in the upcoming month) or precariously housed (i.e., housed in single room occupancy, rooming house, or hotel/motel as a primary residence AND in the past year have a history of 2 or more episodes of being Absolutely Homeless OR one episode of being absolutely homeless of at least 4 weeks duration in the past year); diagnosis of schizophrenia by a psychiatrist based on the Diagnostic and Statistical Manual of Mental Disorders, 4th ed. (DSM-IV-TR) criteria [25]; and the ability to speak French. The exclusion criteria was a reduced capacity to consent [26]. Patients were evaluated at baseline (t0), and a subsample was retested at 6 months to explore sensitivity to change (t1). 

### Procedure

Patients were recruited by mobile mental health outreach teams over a 12 month-period. The mobile mental health outreach teams included a psychiatrist, a nurse and a social worker and were created in France in 2005 to ensure medical, psychiatric and social care for “hard to reach” homeless populations with and without severe mental disorders [27]. The professionals worked on the streets to identify and help people sleeping rough in these four cities. They provided help with immediate needs (e.g., food, access to medical care, and emergency shelter) and ongoing support to assess and address other needs, such as mental health, drug or alcohol problems. All patients that were met by a member of the team were referred to a trained research assistant who checked the eligibility criteria within 24 hours of referral. The results from the Mini International Neuropsychiatric Interview were used to confirm the diagnosis of schizophrenia given by a psychiatrist [28]. The assistant then described the study, responded to any questions the patients had and obtained written informed consent. Evaluations were performed during face-to-face interviews by the research assistant in the offices of the mobile mental health outreach teams, which were located in the downtown area of each city. Participants received twenty-one euros of food coupons for each interview. The patient completed the QoL questionnaire independently or asked for assistance to complete all or part of the questionnaire. All data were collected using netbook computer-assisted interviewing (small inexpensive laptop computers) and transferred to a highly secure central database without using the internet (EpiConcept®).

### Data collection

The following data were collected from patients:

1. Socio-demographic information: gender, age and marital status.

2. Clinical characteristics: Mental Health was assessed using the Modified Colorado Symptom Index (MCSI), which has been validated in homeless individuals [29]. The MCSI contains 14 items that ask about how often in the past month an individual has experienced a variety of mental health symptoms, including loneliness, depression, anxiety, and paranoia. An index score for this scale is calculated by summing each response. Higher scores indicate a higher likelihood of mental health problems. Recovery was assessed using the Recovery Assessment Scale (RAS) [30], which measures various aspects of recovery from the perspective of the consumer, with a particular emphasis on hope and self-determination. This self-administered instrument consisted of 24 items that explore five domains, which are as follows: personal confidence and hope; willingness to ask for help; goal and success orientation; reliance on others; and lack of domination by symptoms. A higher score indicated better recovery. 

 3. Quality of life was assessed using the S-QoL 18 [24] and the Medical Outcomes Study 36-item Short Form Health Survey (SF-36) [31] questionnaires. The S-QoL 18 is a self-administered, multidimensional questionnaire developed and validated for the specific assessment of quality of life in patients with schizophrenia [13,24]. Because the accuracy and completeness of answers to QoL questionnaires may depend on the questionnaires' difficulty and length [32], we have chosen the S-QoL 18 which presents several important properties: the S-QoL 18 is a well-validated questionnaire based exclusively on patients' perspectives, ensuring a more appropriate content than questionnaires based on experts' determination. The items of the S-QoL 18 refer to the present time with one response option, which may be easier for individuals with schizophrenia to understand. Finally, due to its short format, the S-QoL 18 appears better adapted to schizophrenia populations, because of the difficulties in concentration and perception faced by patients with deficit syndrome or thought disorders. The S-QoL 18 consisted of 18 items that describe 8 dimensions, which are as follows (table S1): psychological well-being (PsW), self-esteem (SE), family relationships (RFa), relationships with friends (RFr), resilience (RE), physical well-being (PhW), autonomy (AU) and sentimental life (SL). From these items, a total score (index) was determined. The SF-36 is a self-administered questionnaire consisting of 36 items describing 8 dimensions, which are as follows: Physical Functioning (PF); Social Functioning (SF); Role-Physical Problems (RPP); Role-Emotional Problems (REP); Mental Health (MH); Vitality (VIT); Bodily Pain (BP); and General Health (GH). Two composite scores were calculated, the physical composite score (PCS) and the mental composite score (MCS). Dimension, index and composite scores ranged from 0, indicating the lowest quality of life, to 100, indicating the highest quality of life. 

### Statistical analyses

Statistical analyses were performed to explore the internal structure, reliability, external validity and sensitivity to change of the S-QoL 18. Descriptive statistics of the sample included frequencies and percentages of categorical variables and the means and standard deviations of continuous variables. 

The structure of the S-QoL 18 was explored using confirmatory factor analysis (LISREL model). The following indicators were required: the Root Mean Square Error of Approximation (RMSEA) is acceptable if <0.08 and satisfactory if <0.05, the Comparative Fit Index (CFI) and the General Fit index (GFI) are higher than 0.9, and the Standardised Root Mean Square Residual (SRMR) is closer to 0. The uni-dimensionality of each dimension was assessed using a Rasch analysis. Item-internal consistency was assessed by correlating each item with its scale (corrected for overlap) using Pearson’s coefficient (correlation of 0.4 recommended for supporting item-internal consistency [33]); item discriminant validity was assessed by determining the extent to which items correlate more highly with the dimensions they are hypothesised to represent than with the other ones [34]. For each dimension scale, internal consistency reliability was assessed using Cronbach’s alpha coefficient (a coefficient of at least 0.7 was expected for each scale [33]). Floor and ceiling effects were reported assessing the homogeneous repartition of the response distribution. The goodness-of-fit statistics (INFIT, ranging between 0.7 and 1.3) ensured that all items of the scale measured the same concept. Differential item functioning (DIF) analyses were performed to see whether all items behave in the same way in homeless and non-homeless patients from the validation sample of the S-QoL 18 [24] . 

To explore external validity, Pearson’s correlation coefficients were used to investigate relationships between dimensions of the S-QoL 18 and age, SF-36, MCSI and RAS; dimension scores of the S-QoL 18 were compared across patient groups (i.e., gender and marital status) using Mann-Whitney tests. Several hypotheses were formulated, which are as follows: the S-QoL 18 dimension scores (1) should not differ based on socio-demographic characteristics (i.e., age and gender); (2) should be moderately correlated to the SF-36; (3) should be negatively correlated with the severity of the disease (MCSI) and positively correlated to recovery (RAS). 

Sensitivity to change was assessed at 6 months on a half of the sample (the first 115 patients evaluated at baseline). Patients were classified into the following two groups: one in which health status improved (reduced total MCSI≥20%) and one in which health status did not improve (reduced total MCSI<20%) between inclusion in the study (t0) and the last evaluation at six months (t1). Effect sizes were computed. According to Samsa et al. [35], an effect size of at least 0.2 is recommended as the standard for supporting sensitivity to change. Scores were also compared using paired t-tests.

Acceptability of measuring QoL was tested using the following indicators: proportion of spontaneous refusals of homeless people to answer the S-QoL18 and the percentage of missing values for the S-QoL 18. Direct observation of the research assistants provided complementary information (i.e. commentaries and reactions of patients during the QoL assessment) [36,37]. 

Finally, the QoL of homeless patients was compared with 236 non-homeless French age- (± 2 years) and sex-matched (from the validation sample of the S-QoL 18 [24]) patients using Mann-Whitney tests. 

Data analyses were performed using the PASW 17.0.2 and LISREL software.

### Ethical Approval

The study was carried out in accordance with the principles of the Declaration of Helsinki, 6th revision [38]. We received written consent from all the participants of our study. The Local Ethics Committee (Comité de Protection des Personnes Sud-Méditerranée V, France: trial number 11.050) and the French Drug and Device Regulation Agency (trial number 2011-A00668-33) approved this study. 

## Results

### Sample characteristics

Of the 249 eligible patients, 236 (94.8%) completed the S-QoL 18. The mean age of the sample was 37.9 years (standard deviation = 10.8); in addition, 86.9% of the sample were male and 94.0% were single. The patients’ clinical characteristics (MCAS, MCSI and RAS) and SF-36 scores are presented in table 1. The mean dimension/index scores of the S-QoL 18 among homeless patients are provided in table 2.

**Table 1:** Socio-demographic and clinical characteristics of the study sample.

		**N =236**	**MV^1^**	**Validation Sample S-QoL 18 matched by age and gender N=236**
**1. Sociodemographics**		**N (%)**	**%**	**N (%)**
Gender	Men	205 (86.9)	0.0	205 (86.9)
Age (years)	M (SD)2	37.9 (10.8)	0.0	37.9 (10.7)
Marital status	Single	218 (94.0)	1.7	NA6
**2. Clinical characteristics**		M(SD)**^2^**	**%**	
MCSI3	Total score	21.6 (12.4)	18.6	NA
RAS4	Personal confidence and hope	31.7 (6.5)	20.8	NA
	Willingness to ask for help	10.0 (2.7)	8.9	
	Goal and success orientation	19.4 (3.8)	7.2	
	Reliance on others	13.6 (4.1)	12.3	
	No domination by symptoms	8.9 (3.2)	11.0	
SF-365	Physical Functioning	82.7 (26.4)	0.04	79.1 (22.1)
	Social Functioning	52.7 (33.5)	0.12	54.4 (29.0)
	Role—Physical Problems	74.6 (30.2)	0.12	43.9 (35.6)
	Role—Emotional Problems	55.8 (33.4)	0.12	39.6 (41.7)
	Mental Health	48.2 (22.0)	0.12	57.2 (20.1)
	Vitality	44.0 (20.1)	0.12	48.4 (19.0)
	Bodily Pain	65.6 (32.6)	0.12	64.7 (27.0)
	General Health	54.4 (21.0)	0.12	54.8 (20.1)
	Mental composite score	34.7 (12.5)	0.12	37.3 (11.1)
	Physical composite score	51.4 (11.1)	0.12	46.9 (7.9)

1 MV: missing values ^2^; M (SD): mean (standard deviation) ^3^; MCSI: Modified Colorado Symptom Index ^4^; RAS: Recovery Assessment Scale ^5^; SF-36: Medical Outcomes Study 36-item Short Form Health Survey ^6^; NA: Not available.

**Table 2:** Dimension characteristics of the S-QoL 18 (N=236).

Dimension/Index	M (SD)^1^	Missing values (%)	Item internal consistency min-max	Item discriminant validity min-max	Floor (%)	Ceiling (%)	Alpha2	Infit^3^ min-max
PsW (0-100)	58.4 (29.4)	0.02	0.50-0.54	0.05-0.51	15.0	28.6	0.73	0.90-1.09
SE (0-100)	50.2 (26.8)	0.00	0.48	0.17-0.45	17.7	5.8	0.65	0.94-1.05
RFa (0-100)	34.1 (30.2)	0.04	0.69	0.05-0.44	36.6	2.2	0.81	0.97-0.99
RFr (0-100)	41.6 (31.0)	0.05	0.67	0.15-0.45	26.7	4.8	0.80	0.98-1.00
RE (0-100)	55.6 (23.6)	0.00	0.38-0.48	0.12-0.44	14.3	12.4	0.65	0.91-1.04
PhW (0-100)	49.6 (28.2)	0.00	0.69	0.19-0.43	16.3	5.0	0.84	0.99-1.00
AU (0-100)	60.5 (27.8)	0.00	0.77	0.20-0.41	11.8	8.4	0.88	0.98-0.99
SL (0-100)	31.3 (26.8)	0.01	0.42	0.10-0.49	36.3	3.4	0.60	0.98-1.00
Index (0-100)	47.2 (18.3)	0.10	NA4	NA4	NA4	NA4	NA4	NA4

S-QoL 18-PsW: psychological well-being; SE: self-esteem; RFa: family relationships; RFr: relationships with friends; RE: resilience; PhW: physical well-being; AU: autonomy; and SL: sentimental life.

Scores range from 0 to 100; higher scores represent higher QoL.

1 M (SD): mean (standard deviation) ^2^; Alpha: Cronbach’s alpha ^3^; Infit: Rasch statistics ^4^; NA: non-applicable.

### Construct validity, internal structural validity and reliability

All of the details are provided in table 2. 

The eight-factor structure of the S-QoL 18 was confirmed by confirmatory factor analysis. All of the indices from the confirmatory LISREL model were satisfactory (RMSEA = 0.035, CFI = 0.95, GFI = 0.99 and SRMR = 0.015). The overall scalability was satisfactory. All of the items showed a good fit for the Rasch model in each dimension, and none of the items had a statistical INFIT outside the range of acceptability. Item internal consistency was satisfactory for all dimensions, and each item achieved the 0.40 standard (ranging from 0.42 to 0.77), except for the RE dimension. The correlation of each item with its contributive dimension was higher than that with the other dimensions for 5 of the 8 dimensions (item discriminant validity), which are as follows: SE, RFa, RFr, PhW and AU. Cronbach's alpha coefficients were higher than 0.70 (from 0.73 to 0.88), indicating satisfactory reliability, with the exception of 3 dimensions, which were higher than 0.60 (SE, RE and SL). Floor effects were less than 20%, except for the RFa, RFr and SL. Ceiling effects ranged from 2.2 to 12.4%, with the exception of the PsW. Based on the definition of the DIF, all items behaved in the same way between homeless and non-homeless patients. 

#### External validity

All of the details are provided in table 3. 

**Table 3:** Association between S-QoL 18 dimension scores and gender, marital status, occupational status, health coverage, age, MCAS score, MCIS score, RAS scores and SF-36 scores.

	PsW	SE	RFa	RFr	RE	PhW	AU	SL	Index
Gender M (SD)^1^									
*Male*	58.4 (29.0)	50.1 (27.0)	35.8 (30.3)	42.9 (31.2)	55.5 (24.1)	49.9 (28.0)	61.0 (27.5)	31.4 (26.1)	47.6 (18.3)
*Female*	58.1 (32.2)	51.3 (26.3)	22.9 (27.9)	33.5 (29.3)	56.5 (22.6)	47.2 (29.5)	59.3 (30.3)	30.8 (31.1)	44.5 (18.3)
*P*	0.948	0.825	**0.029**	0.117	0.835	0.612	0.753	0.924	0.388
Marital status (single) M (SD)									
*Yes*	58.9 (29.6)	50.9 (26.2)	35.0 (30.5)	41.7 (30.5)	55.6 (24.0)	50.3 (27.8)	60.7 (27.2)	30.7 (26.3)	47.4 (18.1)
*No*	49.4 (25.8)	41.1 (34.1)	20.5 (22.3)	40.4 (40.2)	54.2 (23.1)	41.1 (35.8)	64.3 (37.6)	43.8 (32.4)	44.6 (23.3)
*P*	0.161	0.165	0.089	0.816	0.906	0.247	0.269	0.112	0.813
Age: r^2^	0.05	-0.01	-0.15	-0.21	-0.14	-0.18	0.02	-0.06	-0.12
MCSI^3^ : r	-0.56**	-0.61**	-0.17*	-0.25**	-0.20**	-0.39**	-0.37**	-0.27**	-0.55**
RAS^4^: r									
*Personal confidence and hope*	0.31**	0.54**	0.27**	0.36**	0.54**	0.50**	0.45**	0.35**	0.64**
*Willingness to ask for help*	0.29**	0.37**	0.13	0.20**	0.38**	0.19**	0.32**	0.22**	0.43**
*Goal and success orientation*	0.07	0.33**	0.10	0.23**	0.50**	0.19**	0.27**	0.14*	0.35**
*Reliance on others*	0.19**	0.31**	0.32**	0.43**	0.36**	0.28**	0.36**	0.30**	0.51**
*No domination by symptoms*	0.20**	0.38**	0.11	0.07	0.23**	0.16**	0.14*	0.20**	0.31**
SF-36^5^: r									
*Physical Functioning*	0.22**	0.22**	0.14*	0.20**	0.18**	0.43**	0.07	0.17*	0.30**
*Social Functioning*	0.45**	0.50**	0.17*	0.26**	0.22**	0.38**	0.29**	0.19**	0.48**
*Role—Physical Problems*	0.26**	0.27**	0.17*	0.18**	0.13	0.54**	0.03	0.20**	0.34**
*Role—Emotional Problems*	0.45**	0.52**	0.15*	0.24**	0.25**	0.47*	0.31**	0.22**	0.50**
*Mental Health*	0.43**	0.58**	0.18**	0.34**	0.22**	0.44**	0.24**	0.27**	0.53**
*Vitality*	0.33**	0.38**	0.17*	0.29**	0.28**	0.52**	0.17*	0.19**	0.45**
*Bodily Pain*	0.26**	0.31**	0.14*	0.22**	0.17**	0.53**	0.02	0.17*	0.35**
*General Health*	0.29**	0.44**	0.15**	0.28**	0.16*	0.56**	0.07	0.20**	0.42**
*Mental composite score*	0.46**	0.58**	0.16*	0.29**	0.25**	0.38**	0.33**	0.23**	0.52**
*Physical composite score*	0.12	0.12	0.12	0.15*	0.10	0.47**	-0.08	0.12	0.21**

S-QoL 18-PsW: psychological well-being; SE: self-esteem; RFa: family relationships; RFr: relationships with friends; RE: resilience; PhW: physical well-being; AU: autonomy; and SL: sentimental life.

1 M (SD): mean (standard deviation) ^2^; r: Pearson correlation coefficient ^3^; MCSI: Modified Colorado Symptom Index ^4^; RAS: Recovery Assessment Scale ^5^; SF-36: Medical Outcomes Study 36-item Short Form Health Survey

* p < 0.05.

** p < 0.01.

The S-QoL 18 index was significantly correlated with all of the SF-36 dimension scores (r=0.21-0.53, all p-values<0.01). As expected, the correlations were weak for RFa, RFr, RE, AU and SL dimensions of the S-QoL 18, which were not assessed by SF-36. The PsW and SE dimensions of the S-QoL 18 showed medium to high correlations with the psychological-like dimensions of the SF-36 (i.e., SF, REP, MH and MCS). In the same way, the PhW dimension of the S-QoL 18 showed medium to high correlations with the physical dimensions of the SF-36 (i.e., PF, RPP, VIT, BP and PCS). 

As expected, S-QoL 18 dimension scores did not reveal any statistical significant link with gender, age and marital status (all p-value > 0.05), except for one dimension and gender (RFa - male 35.8 (SD = 30.3) vs. female 22.9 (SD = 27.9), p=0.029). 

Higher QoL levels were globally associated with lower levels of clinical severity and improved recovery. All of the S-QoL 18 dimension scores were negatively correlated with the MCSI, with correlations scores ranging from -0.61 to -0.20, except for the RFa dimension (r = -0.17). The highest correlations involved the psychological dimensions (i.e., PsW=-0.56 and SE=-0.61; p<0.01) and the index (-0.55; p<0.01). The S-QoL 18 index was significantly correlated with all of the RAS dimension scores (r=0.31-0.64, all p-values<0.01). The S-QoL 18 dimension scores were more highly correlated with RAS scores when the same types of domains were assessed: SE and RE were correlated to personal confidence/hope and willingness to ask for help, RE was correlated with goal and success orientation, and RFr was correlated with reliance on others. 

#### Sensitivity to change

Of the 115 patients who were expected to be retested at 6 months, 90 were evaluated (78.3%). Thirty-seven (41.1%) patients were improved and 53 (58.9%) were not improved based on the MCSI cut-off. Of patients whose health status improved at 6 months, significant improvements between baseline and 6 months (p<0.05) were found for 4 dimensions (PsW, SE, RFa and SL) and the index. S-QoL scores showed an ES higher than 0.2 for 6 dimensions (PsW, SE, RFa, RFr, RE and SL) and for the index score (table 4). To the contrary, of the non-improved patients, no significant differences were found between baseline and 6 months, except for the SL dimension with an ES higher than 0.2. 

**Table 4:** Effect size and paired comparison of S-QoL 18 scores among improved and non-improved patients.

	Baseline	Six months	Paired t test	Effect size2
	M (SD)^1^	M (SD)^1^		
Improved patients N = 37				
PsW	61.2 (30.8)	74.0 (25.1)	p = 0.001	0.45
SE	51.0 (28.8)	60.1 (18.6)	p = 0.044	0.35
RFa	36.8 (29.4)	50.7 (28.5)	p = 0.008	0.45
RFr	51.1 (35.0)	58.6 (26.4)	p = 0.215	0.23
RE	52.7 (24.8)	58.1 (23.4)	p = 0.247	0.24
PhW	53.0 (29.1)	56.4 (24.9)	p = 0.364	0.12
AU	65.5 (27.4)	69.6 (16.5)	p = 0.331	0.15
SL	33.4 (27.0)	45.3 (25.6)	p = 0.017	0.44
Index	49.7 (20.6)	58.7 (17.1)	p = 0.004	0.50
Non-improved patients N = 53				
PsW	60.8 (28.0)	56.8 (32.8)	p = 0.285	-0.14
SE	49.5 (24.5)	47.1 (26.0)	p = 0.536	-0.09
RFa	33.6 (32.0)	34.8 (29.0)	p = 0.812	0.04
RFr	41.6 (30.7)	42.3 (29.3)	p = 0.879	0.02
RE	56.2 (21.2)	53.5 (24.0)	p = 0.517	-0.12
PhW	46.9 (27.7)	45.0 (26.2)	p = 0.637	-0.07
AU	60.8 (26.7)	53.8 (29.0)	p = 0.131	-0.26
SL	26.9 (27.2)	37.3 (28.3)	p = 0.031	0.38
Index	46.8 (16.0)	46.4 (19.7)	p = 0.889	-0.02

S-QoL 18-PsW: psychological well-being; SE: self-esteem; RFa: family relationships; RFr: relationships with friends; RE: resilience; PhW: physical well-being; AU: autonomy; and SL: sentimental life.

1 M (SD): mean (standard deviation) ^2^; Effect size = (Mean score 6 months - _Mean score Baseline) / Standard deviation Baseline of the whole sample.

#### Acceptability of measuring QoL

At the baseline, the percentage of patients who did not complete the QoL questionnaire was 5.2% (13/249), and the missing data for the different dimensions of the S-QoL 18 never exceeded 5%. 

According to research assistants, the homeless were globally satisfied to be receiving a QoL assessment. The questionnaire seemed to be considered by homeless individuals as a basis for discussion of topics they considered important and were too rarely explored by professionals. Homeless individuals reported that three domains were not assessed by the S-QoL 18, which are as follows: 1) administrative and financial problems which prevent them from accessing social and housing aids, 2) living conditions and housing and 3) problems related to the “communication life area” especially when their native language was not French.

#### Comparison of QoL levels between homeless and non-homeless people with schizophrenia

**Figure 1:**
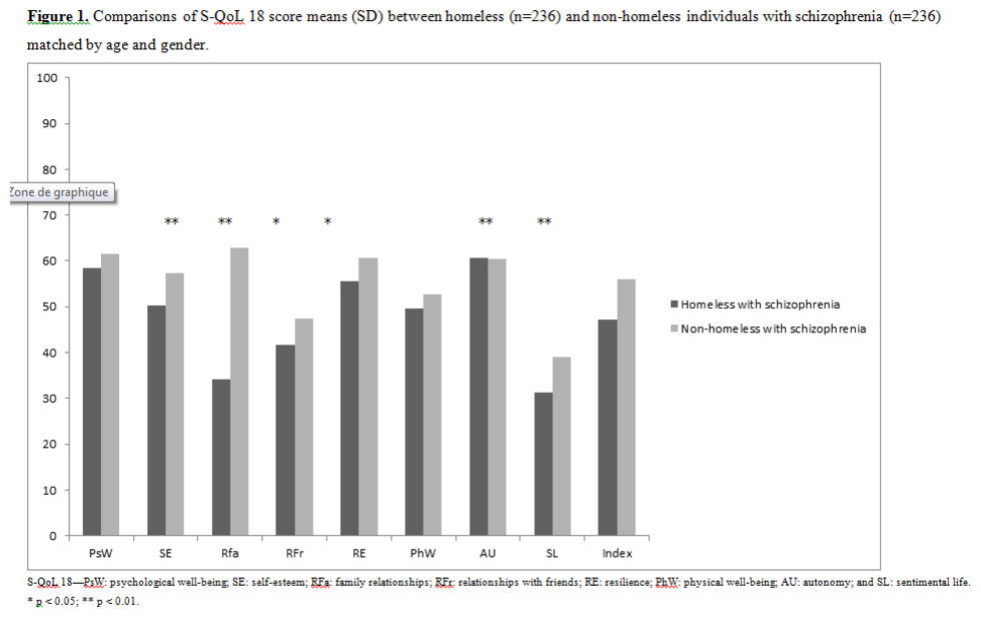
Comparisons of S-QoL 18 score means (SD) between homeless (n=236) and non-homeless individuals with schizophrenia (n=236) matched by age and gender. S-QoL 18—PsW: psychological well-being; SE: self-esteem; RFa: family relationships; RFr: relationships with friends; RE: resilience; PhW: physical well-being; AU: autonomy; and SL: sentimental life. * p < 0.05; ** p < 0.01.

The mean dimension/index scores of the S-QoL 18 were compared with French age- and sex-matched non-homeless patients (age = 37.9 years ± 10.7; 205 men) in Figure 1. All of the S-QoL 18 dimension and index scores were lower in homeless patients than in non-homeless patients with schizophrenia. These differences were significant for 5 dimensions (SE, RFa, RFr, RE and SL) and the index score.

## Discussion

The assessment of QoL has received an increasing amount of attention as an outcome parameter in schizophrenia research; however, whether the QoL measurement is relevant among homeless patients with schizophrenia and to what extent it remains valid in this context are major considerations. Consistent with two previous studies [19,20], our results provide evidence to support the conclusion that homeless patients with schizophrenia answer the QoL questionnaire reliably and consistently with satisfactory psychometric properties. 

With regard to construct validity, the confirmatory factor analysis showed that the structure performed among the homeless individuals matched the initial structure of the S-QoL 18. The unidimensionality of each of the 8 dimensions was supported by satisfactory INFIT statistics. Item internal consistency was satisfactory for all dimensions, with the exception of 1 dimension. Item discriminant validity was less satisfactory (5 among 8 dimensions). However, the overlap was particularly low for the 4 other dimensions (PsW, RE and SL). Cronbach's alpha coefficients were satisfactory for 5 of 8 dimensions (> 0.70). However, concerning the 3 other dimensions (SE, RE and SL), Cronbach's alpha coefficients were acceptable (>0.60). Floor and ceiling effects were also globally acceptable compared to the initial reference population. Finally, no difference was found for item functioning, independent of the homelessness status. Although the properties of the instrument were less satisfactory in our severely ill population (high need level, higher severity of schizophrenia, poor literacy and drug and alcohol intoxication) than in the general population with schizophrenia, these properties were still acceptable according to general standards [24]. 

External validity, which was explored by using socio-demographic characteristics and established psychiatric and QoL measures, globally confirmed our assumptions. As expected, we did not find any association between age and QoL consistent with the results of previous studies [15]. In our study, however, women had lower QoL scores than men for the Relationships with Family dimension. This finding is in accordance with the validation study [24]. We found that S-QoL scores were negatively correlated with the severity of symptoms (MCSI). However, these correlations were moderate in strength, stressing the particular interest of relying on QoL measurement to complement the clinical approach, which does not encompass the entire experience of patients facing chronic illness. Finally, the relationships between QoL and recovery confirm results from previous studies [39]. The correlations were logically moderate because recovery can be considered to be a combination of both objective clinical recovery (i.e., symptomatology and functioning) and subjective recovery (i.e., QoL). 

Another important finding is the acceptability of the S-QoL 18. The rate of missing data, less than 5% for all the dimension scores, and the proportions patients who did not complete QoL questionnaire, 5.2%, were particularly low. Several explanations can be proposed. The S-QoL 18 is one of the shortest instruments among recent QoL measures for use in schizophrenia [24]. According to several authors, a short form of the scale is frequently associated with improved acceptability [40]. Moreover, the content of the S-QoL 18 may be particularly relevant for patients with schizophrenia because its content was ensured by the initial development of the items in the S-QoL 41 on the basis of in-depth interviews with patients suffering from schizophrenia [13]. However, the qualitative approach stressed that some domains were not assessed by the S-QoL 18, suggesting that appropriate measures of these areas should be added to the S-QoL18. In line with our findings, a recent qualitative study conducted with 140 homeless individuals confirmed that administrative and financial problems and living conditions or housing are areas of great importance in QoL of homeless [41]. More globally, this finding raises the problem of loss of information from short-form measures that privilege practical considerations and acceptability (i.e. less constraints on respondent burden), calling the development of computerized adaptive testing to find the accuracy of long-form measures in the years to come [42].

The final property of the S-QoL 18 was its sensitivity to change. This property is of great importance for clinical follow-up and also for the evaluation of interventional studies among homeless individuals. The Index and 4 dimensions were significant over a six-month period and an ES>0.2 were found for 6 dimensions and the index. S-QoL 18 can thus be considered efficient to detect sensitivity to change (of note, this property is rarely assessed in other QoL questionnaires), confirming previous findings on the S-QoL [13,24]. This finding is in accordance with previous studies that suggest specific instruments such as the S-QoL 18 are better able to detect small but clinically relevant improvements [17]. Concerning the two dimensions which were not significant (i.e., ES < 0.2; PhW and AU), the changes were, however, more important than those in the non-improved group. Moreover, we may hypothesize that a significant improvement of these two dimensions requires a period longer than 6 months.

 Finally, a last important result from our study is that homeless patients with schizophrenia experienced particularly low QoL levels in comparison with non-homeless patients across multiple QoL dimensions (SE, RFa, RFr, RE and SL). We may hypothesize that being homeless worsens the QoL in persons with schizophrenia who already have particularly low QoL levels, suggesting that homelessness amongst patients with schizophrenia is a double jeopardy. This hypothesis should be confirmed using longitudinal data. Moreover, we may hypothesize that healthcare and housing management in France for homeless people with mental disorders is not sufficient, despite the numerous available services (e.g., universal health coverage, mobile mental health outreach teams, social assistances, and housing, among others). 

### Limitations and perspectives

Although our findings are striking, some limitations of this study must be considered. 

Even with the large overall sample size of this multi-centre study, the sample may not have been representative of the homeless population with schizophrenia. Because our study took place in large cities, our findings may not generalise to homeless people living in smaller cities in which life conditions and needs may be different. However, our study included southern and northern cities, thus taking into consideration socio-economic, cultural and climatic differences.

Second, validity is considered present when the measurement predicts an external criterion based on a gold standard. In the case of QoL, there is no gold standard and the instrument is considered valid if it consistently fits a series of related construct. In our study, we made comparisons with other measures of quality of life (SF-36), symptomatology (MCSI) and recovery (RAS). Although this choice can be debatable, it can be assumed that our assumptions based on the relationships between the S-QoL 18 and these 3 scales are both reasonable and pragmatic.

Third, sensitivity to change should be further explored. Indeed, despite our encouraging results, a methodological problem remains in the definition of clinical improvement for patients with schizophrenia using the MCSI. Because there is no generally accepted cut-off for the MCSI, we chose an arbitrary cut-off based on the one usually used for the PANSS total score (20%) [43]. Sensitivity to change should thus be assessed using other cut-offs, and other methods should be used to define clinical improvement. However, given the magnitude of the effect sizes in our study, it is arguable that our results would remain satisfactory.

Fourth, an important perspective of our work would be to determine whether our findings depend on site, especially because the study procedures were not standardized between sites. This issue should be explored in future studies on larger samples using in particular recent suitability indices proposed to compare psychometric properties between samples [21,22,32].

Finally, important data concerning external validity were not collected in our study because some assessments are too complex to conduct in this population (e.g., the determination of disease duration and other clinical scales, such as the Positive and Negative Syndrome Scale for symptomatology). Future studies should specifically address these issues.

## Conclusion

These results support the validity, reliability and sensitivity to change of the S-QoL 18 for evaluating QoL in homeless patients suffering from schizophrenia. These data confirm that the S-QoL 18 can provide a useful means, in addition to conventional outcome measures, for assessing/monitoring needs and health in this challenging and understudied population. The assessment of QoL could thus be more widely implemented without concerns regarding the adequacy of using such assessment tools in homeless patients with schizophrenia. 

## Supporting Information

Table S1 **List of S-QoL 18 items.**
(DOCX)

## References

[1] Cougnard A, Grolleau S, Lamarque F, Beitz C, Brugère S et al. (2006) Psychotic disorders among homeless subjects attending a psychiatric emergency service. Soc Psychiatry Psychiatr Epidemiol 41: 904-910. doi:10.1007/s00127-006-0109-4. PubMed: 16924397. 16924397

[2] Fazel S, Khosla V, Doll H, Geddes J (2008) The prevalence of mental disorders among the homeless in western countries: systematic review and meta-regression analysis. PLOS Med 5: e225. doi:10.1371/journal.pmed.0050225. PubMed: 19053169. 19053169 PMC2592351

[3] van der Plas AG, Hoek HW, van Hoeken D, Valencia E, van Hemert AM (2012) Perceptions of quality of life and disability in homeless persons with schizophrenia and persons with schizophrenia living in non-institutional housing. Int J Soc Psychiatry 58: 629-634. doi:10.1177/0020764011419056. PubMed: 21878468. 21878468

[4] Folsom D, Jeste DV (2002) Schizophrenia in homeless persons: a systematic review of the literature. Acta Psychiatr Scand 105: 404-413. doi:10.1034/j.1600-0447.2002.02209.x. PubMed: 12059843. 12059843

[5] Rosenheck RA, Resnick SG, Morrissey JP (2003) Closing service system gaps for homeless clients with a dual diagnosis: integrated teams and interagency cooperation. J Ment Health Policy Econ 6: 77-87. PubMed: 14578540. 14578540

[6] Shinn M, Baumohl J, Hopper K (2001) The prevention of homelessness revisited. Anal Soc Issues Public Policy 1: 95–127. doi:10.1111/1530-2415.00006.

[7] Sadowski LS, Kee RA, VanderWeele TJ, Buchanan D (2009) Effect of a housing and case management program on emergency department visits and hospitalizations among chronically ill homeless adults: a randomized trial. JAMA 301: 1771-1778. doi:10.1001/jama.2009.561. PubMed: 19417194. 19417194

[8] Gallagher TC, Andersen RM, Koegel P, Gelberg L (1997) Determinants of regular source of care among homeless adults in Los Angeles. Med Care 35: 814-830. doi:10.1097/00005650-199708000-00007. PubMed: 9268254. 9268254

[9] Henry JM, Boyer L, Belzeaux R, Baumstarck-Barrau K, Samuelian JC (2010) Mental disorders among homeless people admitted to a French psychiatric emergency service. Psychiatr Serv 61: 264-271. doi:10.1176/appi.ps.61.3.264. PubMed: 20194403. 20194403

[10] Kuhn R, Culhane DP (1998) Applying cluster analysis to test a typology of homelessness by pattern of shelter utilization: results from the analysis of administrative data. Am J Community Psychol 26: 207-232. doi:10.1023/A:1022176402357. PubMed: 9693690. 9693690

[11] Goering PN, Streiner DL, Adair C, Aubry T, Barker J et al. (2011) The At Home/Chez Soi trial protocol: a pragmatic, multi-site, randomised controlled trial of a Housing First intervention for homeless individuals with mental illness in five Canadian cities. BMJ Open 1: e000323. doi:10.1136/bmjopen-2011-000323. PubMed: 22102645. PMC3221290 22102645

[12] Shern DL, Tsemberis S, Anthony W, Lovell AM, Richmond L et al. (2000) Serving street-dwelling individuals with psychiatric disabilities: outcomes of a psychiatric rehabilitation clinical trial. Am J Public Health 90: 1873-1878. doi:10.2105/AJPH.90.12.1873. PubMed: 11111259. 11111259 PMC1446423

[13] Auquier P, Simeoni MC, Sapin C, Reine G, Aghababian V et al. (2003) Development and validation of a patient-based health-related quality of life questionnaire in schizophrenia: the S-QoL. Schizophr Res 63: 137-149. doi:10.1016/S0920-9964(02)00355-9. PubMed: 12892868. 12892868

[14] Bobes J, García-Portilla P, Sáiz PA, Bascarán T, Bousoño M (2005) Quality of life measures in schizophrenia. Eur Psychiatry 20 Suppl 3: S313-S317. doi:10.1016/S0924-9338(05)80182-8. PubMed: 16459242. 16459242

[15] Reine G, Simeoni MC, Auquier P, Loundou A, Aghababian V et al. (2005) Assessing health-related quality of life in patients suffering from schizophrenia: a comparison of instruments. Eur Psychiatry 20: 510-519. doi:10.1016/j.eurpsy.2005.05.009. PubMed: 16139488. 16139488

[16] Boyer L, Millier A, Perthame E, Aballea S, Auquier P et al. (2012) Quality of life is predictive of relapse in schizophrenia. BMC Psychiatry 13: 15 PubMed: 23302219. 10.1186/1471-244X-13-15 PMC3544732 23302219

[17] Cramer JA, Rosenheck R, Xu W, Thomas J, Henderson W et al. (2000) Quality of life in schizophrenia: a comparison of instruments. Department of Veterans Affairs Cooperative Study Group on Clozapine in Refractory Schizophrenia. Schizophr Bull 26: 659-666. doi:10.1093/oxfordjournals.schbul.a033484. PubMed: 10993404. 10993404

[18] Balshem H, Christensen V, Tuepker A, Kansagara D (2011) A Critical Review of the Literature Regarding Homelessness among Veterans. VA-ESP Project #05-225 21678634

[19] Garcia-Rea E, LePage JP (2008) Reliability and validity of World Health Organization Quality of Life-100 in homeless substance-dependent veteran population. J Rehabil Res Dev 45: 619-625. doi:10.1682/JRRD.2007.03.0048. PubMed: 18712647. 18712647

[20] Riley ED, Bangsberg DR, Perry S, Clark RA, Moss AR et al. (2003) Reliability and validity of the SF-36 in HIV-infected homeless and marginally housed individuals. Qual Life Res 12: 1051-1058. doi:10.1023/A:1026166021386. PubMed: 14651422. 14651422

[21] Baumstarck K, Pelletier J, Aghababian V, Reuter F, Klemina I et al. (2012) Is the concept of quality of life relevant for multiple sclerosis patients with cognitive impairment? Preliminary results of a cross-sectional study. PLOS ONE 7: e30627. doi:10.1371/journal.pone.0030627. PubMed: 22292002. 22292002 PMC3264575

[22] Baumstarck K, Reuter F, Boucekine M, Aghababian V, Klemina I et al. (2012) Relevance of quality of life assessment for multiple sclerosis patients with memory impairment. PLOS ONE 7: e50056. doi:10.1371/journal.pone.0050056. PubMed: 23239975. 23239975 PMC3519834

[23] Fitzpatrick R, Ziebland S, Jenkinson C, Mowat A (1992) Importance of sensitivity to change as a criterion for selecting health status measures. Qual Health Care 1: 89-93. doi:10.1136/qshc.1.2.89. PubMed: 10136848. 10136848 PMC1054970

[24] Boyer L, Simeoni MC, Loundou A, D'Amato T, Reine G et al. (2010) The development of the S-QoL 18: a shortened quality of life questionnaire for patients with schizophrenia. Schizophr Res 121: 241-250. doi:10.1016/j.schres.2010.05.019. PubMed: 20541912. 20541912

[25] APA (2000) DSM-IV. Diagnostic and Statistical Manual of Mental Disorders, 4th ed. Text revised. Washington, DC: American Psychiatric Association.

[26] Jeste DV, Saks E (2006) Decisional capacity in mental illness and substance use disorders: empirical database and policy implications. Behav Sci Law 24: 607-628. doi:10.1002/bsl.707. PubMed: 16883611. 16883611

[27] Girard V, Sarradon-Eck A, Payan N, Bonin JP, Perrot S et al. (2012) The analysis of a mobile mental health outreach team activity: from psychiatric emergencies on the street to practice of hospitalization at home for homeless people. Presse Med 41: e226-e237. doi:10.1016/j.lpm.2011.09.032. PubMed: 22244723. 22244723

[28] Sheehan DV, Lecrubier Y, Sheehan KH, Amorim P, Janavs J et al. (1998) The Mini-International Neuropsychiatric Interview (M.I.N.I.): the development and validation of a structured diagnostic psychiatric interview for DSM-IV and ICD-10. J Clin Psychiatry 59 Suppl 20: 22-57;quiz: 9881538. 9881538

[29] Conrad KJ, Yagelka JR, Matters MD, Rich AR, Williams V et al. (2001) Reliability and validity of a modified Colorado Symptom Index in a national homeless sample. Ment Health Serv Res 3: 141-153. doi:10.1023/A:1011571531303. PubMed: 11718206. 11718206

[30] Corrigan PW, Salzer M, Ralph RO, Sangster Y, Keck L (2004) Examining the factor structure of the recovery assessment scale. Schizophr Bull 30: 1035-1041. doi:10.1093/oxfordjournals.schbul.a007118. PubMed: 15957202. 15957202

[31] Ware JE Jr., Sherbourne CD (1992) The MOS 36-item short-form health survey (SF-36). I. Conceptual framework and item selection. Med Care 30: 473-483. doi:10.1097/00005650-199206000-00002. PubMed: 1593914. 1593914

[32] Baumstarck K, Boyer L, Boucekine M, Aghababian V, Parola N et al. (2013) Self-reported quality of life measure is reliable and valid in adult patients suffering from schizophrenia with executive impairment. Schizophr Res, 147: 2013 Apr 5 PubMed: 23566495pii: S0920-9964(13)00154-0 101016/jschres201303008. PubMed: 23566495. 10.1016/j.schres.2013.03.008 23566495

[33] Carey RG, Seibert JH (1993) A patient survey system to measure quality improvement: questionnaire reliability and validity. Med Care 31: 834-845. doi:10.1097/00005650-199309000-00008. PubMed: 8366685. 8366685

[34] Campbell DT, Fiske DW (1959) Convergent and discriminant validation by the multitrait-multimethod matrix. Psychol Bull 56: 81-105. doi:10.1037/h0046016. PubMed: 13634291. 13634291

[35] Samsa G, Edelman D, Rothman ML, Williams GR, Lipscomb J et al. (1999) Determining clinically important differences in health status measures: a general approach with illustration to the Health Utilities Index Mark II. Pharmacoeconomics 15: 141-155. doi:10.2165/00019053-199915020-00003. PubMed: 10351188. 10351188

[36] Boulton M, Fitzpatrick R, Swinburn C (1996) Qualitative research in health care: II. A structured review and evaluation of studies. J Eval Clin Pract 2: 171-179. doi:10.1111/j.1365-2753.1996.tb00041.x. PubMed: 9238586. 9238586

[37] Fitzpatrick R, Boulton M (1996) Qualitative research in health care: I. The scope and validity of methods. J Eval Clin Pract 2: 123-130. doi:10.1111/j.1365-2753.1996.tb00036.x. PubMed: 9238582. 9238582

[38] WMA (2008) World Medical Association: Declaration of Helsinki, 6th Revision. Edinburgh.

[39] Roe D, Mashiach-Eizenberg M, Lysaker PH (2011) The relation between objective and subjective domains of recovery among persons with schizophrenia-related disorders. Schizophr Res 131: 133-138. doi:10.1016/j.schres.2011.05.023. PubMed: 21669512. 21669512

[40] McKenna SP (1997) Measuring quality of life in schizophrenia. Eur Psychiatry, 12 Suppl 3: 267s-274s. PubMed: 19698579. 19698579 10.1016/S0924-9338(97)89096-7

[41] Palepu A, Hubley AM, Russell LB, Gadermann AM, Chinni M (2012) Quality of life themes in Canadian adults and street youth who are homeless or hard-to-house: a multi-site focus group study. Health Qual Life Outcomes 10: 93. doi:10.1186/1477-7525-10-93. PubMed: 22894551. 22894551 PMC3462681

[42] Ware JE Jr. (2008) Improvements in short-form measures of health status: introduction to a series. J Clin Epidemiol 61: 1-5. doi:10.1016/j.jclinepi.2007.08.008. PubMed: 18083456. 18083456

[43] Jäger M, Schmauss M, Laux G, Pfeiffer H, Naber D et al. (2009) Early improvement as a predictor of remission and response in schizophrenia: Results from a naturalistic study. Eur Psychiatry 24: 501-506. doi:10.1016/j.eurpsy.2009.02.005. PubMed: 19559572. 19559572

