# Supplementary material for: Toward Meeting the Needs of Homeless People with Schizophrenia: The Validity of Quality of Life Measurement
Source: PLoS One. 2013 Oct 25;8(10):e79677. doi: 10.1371/journal.pone.0079677 (PMC3808288; doi:10.1371/journal.pone.0079677)
Supplement: Table S1 — List of S-QoL 18 items. (DOCX) [file pone.0079677.s001.docx]

**Table S1.** List of S-QoL 18 items.

| N° | Item’s general meaning | Dimensions |
| --- | --- | --- |
|  | At the present time, |  |
| q1 | I am confident in life | Self-esteem |
| q2 | I fight to succeed in my life | Resilience |
| q3 | I am able to plan for my professional or personal future | Resilience |
| q4 | I am in a good mood. I am at ease with myself. | Self-esteem |
| q5 | I feel free to make decisions | Autonomy |
| q6 | I feel free to act | Autonomy |
| q7 | I make efforts to work | Resilience |
| q8 | I am in good physical shape | Physical well-being |
| q9 | I am full of energy | Physical well-being |
| q10 | I am helped and supported by my family | Family relationships |
| q11 | My family pays attention to me | Family relationships |
| q12 | I am helped and supported by my friends or my relatives | Relationships with friends |
| q13 | I have friends | Relationships with friends |
| q14 | I am satisfied with my love life | Sentimental life |
| q15 | I am able to achieve my sentimental projects | Sentimental life |
| q16 | I have difficulty concentrating or thinking straight | Psychological well-being |
| q17 | I feel cut off from the outside world | Psychological well-being |
| q18 | I have difficulty expressing my feelings | Psychological well-being |
